# Molecular Binding Contributes to Concentration Dependent Acrolein Deposition in Rat Upper Airways: CFD and Molecular Dynamics Analyses

**DOI:** 10.3390/ijms19040997

**Published:** 2018-03-27

**Authors:** Jinxiang Xi, Qin Hu, Linlin Zhao, Xiuhua April Si

**Affiliations:** 1 Department of Biomedical Engineering, California Baptist University, Riverside, CA 92504, USA; 2 School of Engineering and Technology, Eastern Michigan University, Ypsilanti, MI 48197, USA; qhu1@emich.edu; 3 The Rutgers Center for Computational and Integrative Biology, Camden, NJ 08102, USA; linlin.zhao@rutgers.edu; 4 Aerospace and Mechanical Engineering, California Baptist University, Riverside, CA 92504, USA; asi@calbaptist.edu

**Keywords:** acrolein, molecular binding, molecular dynamics simulation, concentration-dependent, cigarette smoking

## Abstract

Existing in vivo experiments show significantly decreased acrolein uptake in rats with increasing inhaled acrolein concentrations. Considering that high-polarity chemicals are prone to bond with each other, it is hypothesized that molecular binding between acrolein and water will contribute to the experimentally observed deposition decrease by decreasing the effective diffusivity. The objective of this study is to quantify the probability of molecular binding for acrolein, as well as its effects on acrolein deposition, using multiscale simulations. An image-based rat airway geometry was used to predict the transport and deposition of acrolein using the chemical species model. The low Reynolds number turbulence model was used to simulate the airflows. Molecular dynamic (MD) simulations were used to study the molecular binding of acrolein in different media and at different acrolein concentrations. MD results show that significant molecular binding can happen between acrolein and water molecules in human and rat airways. With 72 acrolein embedded in 800 water molecules, about 48% of acrolein compounds contain one hydrogen bond and 10% contain two hydrogen bonds, which agreed favorably with previous MD results. The percentage of hydrogen-bonded acrolein compounds is higher at higher acrolein concentrations or in a medium with higher polarity. Computational dosimetry results show that the size increase caused by the molecular binding reduces the effective diffusivity of acrolein and lowers the chemical deposition onto the airway surfaces. This result is consistent with the experimentally observed deposition decrease at higher concentrations. However, this size increase can only explain part of the concentration-dependent variation of the acrolein uptake and acts as a concurrent mechanism with the uptake-limiting tissue ration rate. Intermolecular interactions and associated variation in diffusivity should be considered in future dosimetry modeling of high-polarity chemicals such as acrolein.

## 1. Introduction

Acrolein is an unsaturated aldehyde that induces inflammatory responses [1]. It presents in both mainstream and side stream cigarette smoke at levels from 3 to 220 µg per cigarette [2]. The concentration of acrolein at the surface of the respiratory tract of a cigarette smoker can be up to 80 µM [3]. Exposure to acrolein has been linked to a broad spectrum of human diseases afflicting almost every single organ in the body, particularly the pulmonary and cardiovascular tissues [4,5]. Chronic cough and airway hyperreactivity were frequently observed after acrolein exposures [6]. Acrolein is considered a major toxicant contributing to the etiology of cigarette smoke-associated respiratory diseases, such as chronic obstructive pulmonary disease (COPD), emphysema, chronic bronchitis, and asthma [7,8]. Due its high reactivity, significant acrolein deposition has been reported in the upper airways of rats [9,10]. Acrolein can cause cardiovascular distress and increase cardiac arrhythmia risks even at low-level exposures that don’t elicit respiratory distress responses [11]. TRPA1 (Transient Receptor Potential Ankyrin 1), a target of acrolein, has been suggested to be responsible for the local and systemic toxicological injuries after exposures [11]. In human hepatocytes, acrolein triggers endoplasmic reticulum stress and activates eIF2alpha and GADD153 (growth arrest and DNA damage 153), resulting in cell death [12]. Exposure to acrolein was also demonstrated to induce inflammatory responses in middle ear epithelial cells [13].

In mainstream cigarette smoke, the acrolein vapor is generated from burning tobacco and enters the human respiratory tract at a relatively high temperature. This temperature quickly drops within the respiratory tract to the body’s temperature (37.5 °C), which is below the boiling point of acrolein (53 °C). As a result, the kinetic energy of the acrolein will reduce and molecular binding among molecules occurs, leading to larger compounds and, eventually, detectable droplets (i.e., condensation). The larger molecular compound will lead to less diffusivity, which will further lead to reduced deposition rate and different deposition distribution. In sidestream (or second-hand) smoke, acrolein is at the ambient temperature (say 23 °C) and more molecular binding is expected. The concentration of acrolein in sidestream smoke is also lower. In this scenario, the assumption of free acrolein molecules (~0.73 nm) may still be reasonable. Acrolein binding can be influenced by many factors, such as polarity, temperature, concentration, etc., and, therefore, a heterogeneous distribution of acrolein compound sizes is expected. The binding process is a result of the competition between the intermolecular attraction force that keeps the molecules together and the vibrational force that attempts to free the individual molecule from the agglomerate. Any factors that affect these two forces will affect the evolution of the acrolein compound size and the subsequent acrolein deposition.

Due to its high toxicity, there are currently no simple tests available to determine personal exposure to acrolein. Animal studies, in vitro assays, and numerical modeling have been employed as surrogates in investigating acrolein toxicity (1, 2, 5). Morris [9] measured the deposition of acrolein vapor in surgically isolated upper respiratory tracts (URT) of F344 rats and reported a different uptake pattern of acrolein than that of other vapor species. Instead of maintaining a steady uptake rate during the exposure, the URT uptake of acrolein slowly decreased throughout the 40-min exposure period. Struve et al. [10] studied the URT uptake efficiency (UE) in rates under different acrolein concentrations and observed a significant dependence of UE on the exposure concentration, as well as on the flow rate and exposure duration. The UE was measured to be 62%, 38%, and 28% at the exposure concentrations of 2, 10, and 20 µg/L, respectively. It was suggested that the concentration-dependent UE might be related to the respiratory glutathione (GSH) concentration, which decreased with time during a continuous acrolein vapor exposure [10]. However, an explicit explanation of the UE variation with acrolein concentration was not given.

A fundamental question arises in computational modeling of acrolein deposition: Does acrolein within the respiratory tract exist as a gas, vapor, droplets, or as a combination of these? Acrolein is often generated at temperatures higher than its boiling point (53 °C), such as the combustion temperature of a cigarette (800–900 °C) [14] or of fried cooking (150–225 °C) [15]. The initial phase of acrolein is a gas phase; however, both environmental temperature (0–30 °C, with 23 °C in a work environment) and body temperature (37.5 °C) are lower than the boiling point. Therefore, when considering the toxic effect of ambient acrolein on inhalation, the vapor and droplets of liquid phases of acrolein can coexist.

Nearly all previous computational modeling of acrolein deposition have assumed a constant size of the compound, as well as a constant molecular diffusivity. There are good reasons for this assumption, and many challenges exist if the modeling is done otherwise. First, in contrast to a well-defined diameter (and diffusivity) for a single acrolein molecule, the size of the acrolein vapor or droplet for a given scenario is often unknown, even though measurement of the size distribution of such droplet aerosols is possible [16]. Second, if different phases of acrolein, such as gas (single molecules), vapor (small molecule compounds), and droplets (large molecule compounds) co-exist, what are their statistical fractions? Thirdly, acrolein molecules have high polarity and thus exhibit an affinity for neighboring polar molecules. The acrolein binding/breaking process may be dynamic and thus will change the diameters over time. This effect may be more pronounced inside the respiratory tract, where the relative humidity is nearly saturated, and the temperature is 15 °C below the boiling temperature of acrolein. In addition, the chance of molecular binding may be affected by the acrolein concentration, temperature, or pressure. Literature that experimentally quantifies the dynamics of acrolein at ambient or body temperatures is scarce.

In this study, an alternative mechanism that may contribute to the concentration-dependent acrolein deposition is proposed. It is hypothesized that a high concentration of high-polarity acrolein leads to the agglomeration of acrolein–acrolein and/or acrolein–water molecules, which reduces the effective gas diffusivity and leads to lower deposition in the rat airway. This hypothesis will be numerically tested using two approaches. First, we will demonstrate that molecule agglomeration can lead to significant deposition decreases in a rat nose model, which are comparable to those observed in the experiments [9,10], by using computational fluid dynamic (CFD) simulations. The equivalent agglomerate sizes that cause experimentally observed deposition rates will be computed by comparing the CFD predictions to experimental data. Second, we will demonstrate by means of molecular dynamic (MD) simulations that the agglomeration of acrolein/water molecules is feasible at certain concentrations. The probability of molecular agglomeration will be quantified under the influence of different acrolein concentrations.

## 2. Results

### 2.1. Airflow

**Figure 1:**
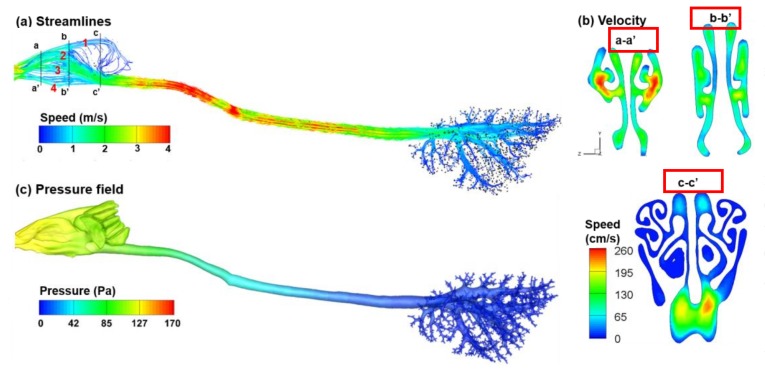
Airflow in the rat airways: (**a**) streamlines; (**b**) cross-sectional velocity distribution in the rat nose; and (**c**) pressure field.

The distribution of inhaled airflow can be highly heterogeneous within the rat nasal cavity (Figure 1). The inhaled airflow splits into four streams after the nostril, with one being ventilated into the olfactory region, one curving up to the dorsal meatus, the third going flat through the middle meatus, and the forth curving downward slightly and passing through the nasal floor (Figure 1a). These four streams merge at the caudal turbinate and enter the trachea at a much higher speed. The rat nasal cavity has considerably larger cross-sectional areas relative to the nostril and trachea, leading to a slow-moving flow through the nasal passages as well as a prolonged residence time for vapor to deposit. The maze-like turbinate region further facilitates the vapor deposition by interception. Moreover, the width of the rat nasal passage is very small (0.1–0.5 mm), making it even easier for vapors to diffuse to the nasal walls. Comparing the flow distributions among the three sections (a-a’, b-b’, and c-c’) reveals an interesting flow evolution within the nasal cavity, which should be associated with the inherent nose functions of air-conditioning, cleaning, and olfaction. In the anterior nose (cross-section a-a’), the main flow concentrates in the two lateral meatuses (i.e., respiratory turbinate) (Figure 1b). The flow is slower and more evenly distributed in the middle nose (cross-section b-b’), indicating an area expansion in the respiratory turbinate, which functions to filter inhaled toxins and warm/humidify inhaled air. In the posterior nose (cross-section c-c’), the flow is much slower in the ethmoidal concha (olfactory region) than in other regions. A higher-speed flow is also observed in the superior ethmoidal concha, which facilitates the convection of inhaled chemicals to the olfactory region (cross-section c-c’). By contrast, low-speed flows are observed in other regions of the superior ethmoidal concha, which may allow longer residence time for olfactory perception of inhaled chemicals.

### 2.2. Deposition Fraction

**Figure 2:**
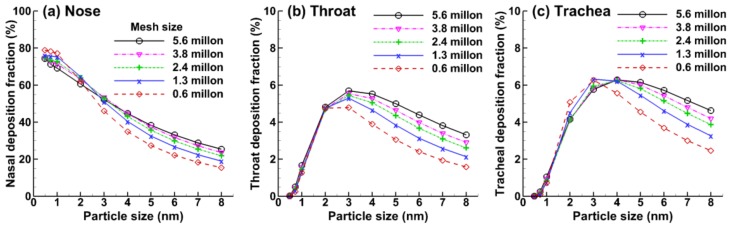
Mesh sensitivity analysis of the predicted sub-regional deposition fractions in (**a**) nose; (**b**) throat; and (**c**) trachea.

To ensure grid-independent results, a grid sensitivity study was performed following Xi et al. [17]. Five grid densities (i.e., 0.6 million, 1.3 million, 2.4 million 3.8 million, and 5.6 million), each with five-layer prismatic meshes in the near-wall region, were tested for the rat airway model. Diffusivities corresponding to molecular compounds in the range of 0.48–8.0 nm were considered. The small molecule size of 0.48 nm and large molecule size of 8 nm were considered for comparison purposes only and may not necessarily represent any physical condition of inhaled acrolein. Deposition fractions (DFs) in three sub-regions, which included the nose, throat, and trachea, were examined. A larger dependence of sub-regional DFs on mesh density was observed for particles larger than 3 nm, with the maximum variation occurring at the largest particle size considered (8 nm), as shown in Figure 2. It is also observed that by varying the mesh size from 3.8 million to 5.4 million, the variation of DFs is insignificant (less than 1%) in all three regions considered, suggesting that the numerical predictions reached grid-independent results in this study. The final mesh for the rat nose model had 3.8 million elements.

As expected, the DF in the rat nose constantly decreased from 0.48 nm to 8 nm because of the decreased aerosol diffusivity. However, a different profile of DF-d_p_ was observed in both the throat and trachea, which increased first and then constantly decreased as particle size increased (diffusivity decreased). The peak value of DF was reached at approximately 3 nm in the throat and at 4 nm in the trachea. The initial increase in DF in the throat and trachea arises from the reduced filtration (scrubbing) of larger molecules by the nose and, therefore, the enhanced availability of acrolein in the throat and trachea. The random motions of molecules in the range of 1–3 nm are still large enough to reach the airway wall of the rat throat and trachea when the vapor molecules travel through these two regions. As particle size continues to increase, the diffusivity may become too low to reach the airway wall even though there is slightly more availability of vapor, thereby leading to a decrease in DF for particles larger than 4 nm (Figure 2b,c).

### 2.3. Surface Deposition Distribution

**Figure 3:**
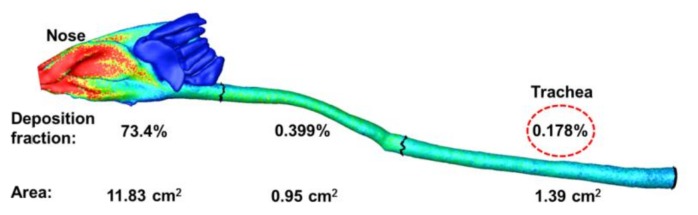
Dimension and deposition fraction of inhaled acrolein in three sub-regions: Nose, throat (pharynx-larynx), and trachea.

The deposition fractions of inhaled acrolein in the three regions of the rat airway (i.e., nose, throat, and trachea) are shown in Figure 3. The surface area of each region is also listed in Figure 3. A fraction of 73.4% of inhaled acrolein was predicted to deposit in the rat nose, which is higher than in the human nose and mouth [18,19]. This is not unexpected, considering that the rat nasal airway is much more complex and smaller than that of humans [20]. The rat nasal cavity is like a maze, with the average width of the nasal passage being less than 0.5 mm [20]. Because of their high diffusivity, it is much easier for inhaled acrolein compounds to reach the rat nasal airway wall via Brownian motion (molecule diffusion) and deposit there. Due to the significant depletion of inhaled acrolein in the rat nose, the deposition fractions in the downstream throat and trachea are much smaller, which are 0.399% and 0.178% (dashed ellipse in Figure 3), respectively. Considering the deposition distribution, inhaled acrolein quickly deposits in the anterior nose, while the deposition fraction diminishes along the respiratory tract. However, very few inhaled acrolein reach the olfactory region, because of the low flow ventilation to this region.

**Figure 4:**
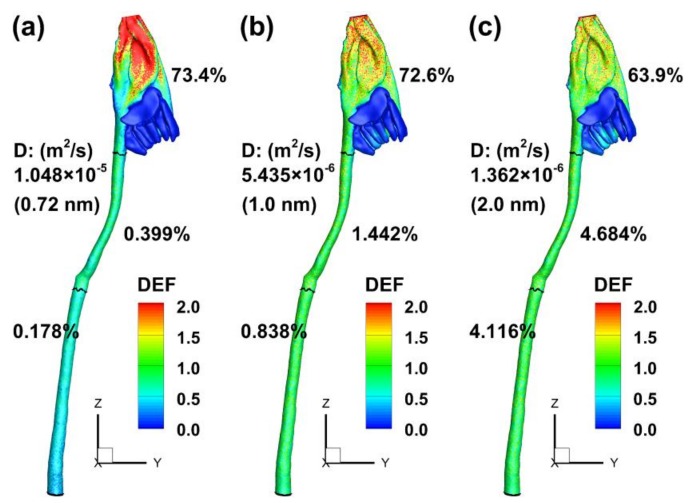
Effect of diffusivity on sub-regional deposition fractions: (**a**) *D* = 1.048 × 10^−5^ m^2^/s; (**b**) *D* = 5.435 × 10^−6^ m^2^/s; and (**c**) *D* = 1.362 × 10^−6^ m^2^/s.

The influence of vapor diffusivity on deposition distribution in the rat airway is shown in Figure 4. The vapor diffusivity considered ranged from 1.048 × 10^−5^ m^2^/s to 1.362 × 10^−6^ m^2^/s. The control case of *D* = 1.048 × 10^−5^ m^2^/s had been adopted in most previous acrolein studies [21] and was used herein to represent the compound size without molecular binding (or equivalently, 0.72 nm in diameter based on the Stokes-Einstein equation as shown in Equation (6). Similarly, the diffusivity of 5.435 × 10^−6^ m^2^/s and 1.362 × 10^−6^ m^2^/s were equivalent to 1.0 nm and 2.0 nm, respectively, which represented two levels of molecular binding. As shown in Figure 4, although the total deposition in the rat respiratory tract remains largely unchanged among the three diffusivities, the deposition distribution is very different, indicating that the local deposition is highly sensitive to diffusivity. A slight decline of DF in the rat nose (from 74.9% to 63.9%) was predicted as the diffusivity decreased from 1.048 × 10^−5^ m^2^/s to 1.362 × 10^−6^ m^2^/s; meanwhile, a dramatic increase in DF was predicted in both the rat throat and trachea. In particular, the tracheal DF increased by nearly two orders of magnitude, from 0.178% at 1.048 × 10^−5^ m^2^/s (0.72 nm) to 4.116% at 1.362 × 10^−6^ m^2^/s (2 nm). This high sensitivity of local deposition makes it elusive to accurately predict the acrolein deposition in the trachea, considering the potential uncertainties in characterizing acrolein diffusivities and aerosol sizes.

### 2.4. Computation vs. In Vitro Experiment

**Figure 5:**
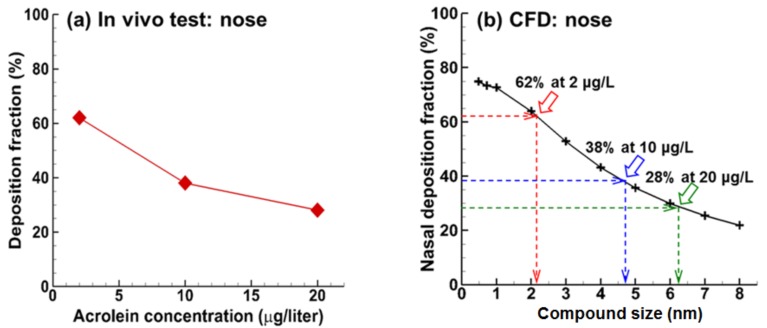
In vivo measured and CFD predicted deposition fractions of acrolein in the rat nose: (**a**) measured acrolein deposition fractions that decreased at higher inhalation concentrations [10] and (**b**) CFD predictions that decreased with decreasing diffusivity (equivalently, increasing particle size converted by the Stokes-Einstein equation, i.e., Equation (6)). The three points (indicated by arrows) in (**b**) correspond to the three measurement points in (**a**).

An interesting phenomenon that has been observed in previous in vitro tests of acrolein exposure in rodents is that the deposition fraction of acrolein in the rat nose decreases at higher concentrations of acrolein [9,10]. Even though the limited reaction rate in the tissue has often been used to explain this deposition, we hypothesize that molecular agglomeration (and decreased diffusivity) may also be a mechanism that contributes to this observation. Molecular binding via Van der Waals forces or molecular binding via hydrogen bonds is possible in light of the high polarity of acrolein and an environment with a temperature lower than the chemical’s boiling point. It is also believed that these molecular interactions will be augmented at higher acrolein concentrations. To match the measured DFs measured by Struve et al. [10] of 62%, 38%, 28% at 2, 10, and 20 µg/L, the required aerosol size should be 2.1, 4.8, and 6.2 nm, respectively (Figure 5). Further studies of the interactions among acrolein molecules are needed to verify this hypothesis. It is also noted that other concurrent mechanisms may also contribute to this observation, such as the reaction of acrolein with glutathione (GSH) on the tissue surface.

### 2.5. Binding Potential Analysis

**Figure 6:**
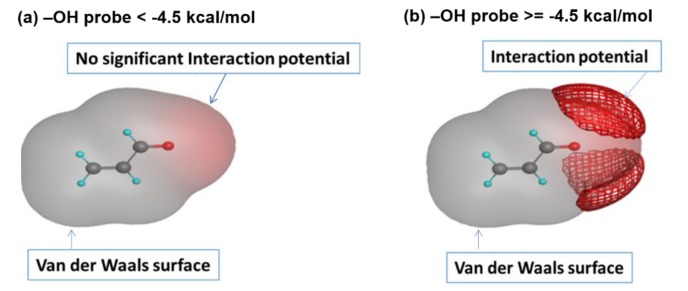
Visualization of Van der Waals surface and interaction potential surface of acrolein: (**a**) –OH probe < −4.5 kca/mol, and (**b**) –OH probe ≥ −4.5 kca/mol.

It is known that an acrolein molecule has a polar negative Van der Waals surface area comparable with that of Dihydrogen oxide and Hydrogen fluoride [22]. This result indicates that an acrolein molecule has polarity, so the intermolecular force (dipole–dipole attractions) can exist among a large number of acrolein molecules. The influence of dipole–dipole attractions on molecular properties has been reported [23,24,25]. Therefore, the dipole–dipole attractions among acrolein molecules also have the potential to influence the acrolein’s molecular properties, such as molecular size. To test this possibility, the Van der Waals surface map and interaction potential surface were calculated for acrolein using the software MOE (Molecular Operating Environment, Chemical Computing Group, Montreal, QC, Canada), as displayed in Figure 6. The interaction potential was calculated using –OH as a probe; the interaction potential disappeared when the –OH probe had an interaction energy less than −4.5 kcal/mol (Figure 6a). However, the interaction potential was significant when we increased the interaction energy of –OH probe (Figure 6b). When the concentration of acrolein was higher, the possibility of intermolecular interactions will increase, which resulted in a higher interaction energy of each molecule, and the influence of dipole-dipole interactions on molecule size could not be neglected anymore.

### 2.6. Molecular Dynamics Simulations

**Figure 7:**
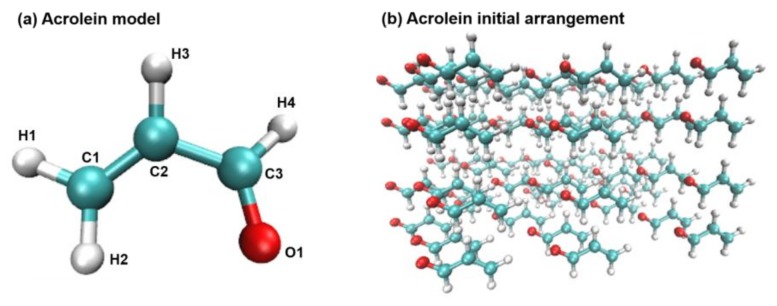
Acrolein model: (**a**) The ball-and-stick model of an acrolein molecule. Color scheme: Hydrogen in white, oxygen in red, carbon in green; (**b**) initial arrangement of 72 acrolein molecules.

Interactions between acrolein molecules were studied using MD simulations. Figure 7 shows the ball-and-stick model of an acrolein molecule, with hydrogen in white, oxygen in red, and carbon in cyan. Initially, 72 acrolein molecules were evenly arranged in space and immersed in two media: water, which has a similar polarity to acrolein, and ethanol, whose polarity is much smaller. Comparison of MD results in the bath of water and ethanol will shed light on the influence of molecular polarity on acrolein binding. Two different numbers of acrolein molecules were simulated being embedded in the same number of water molecules to study the effects of acrolein concentration on molecule interactions.

**Figure 8:**
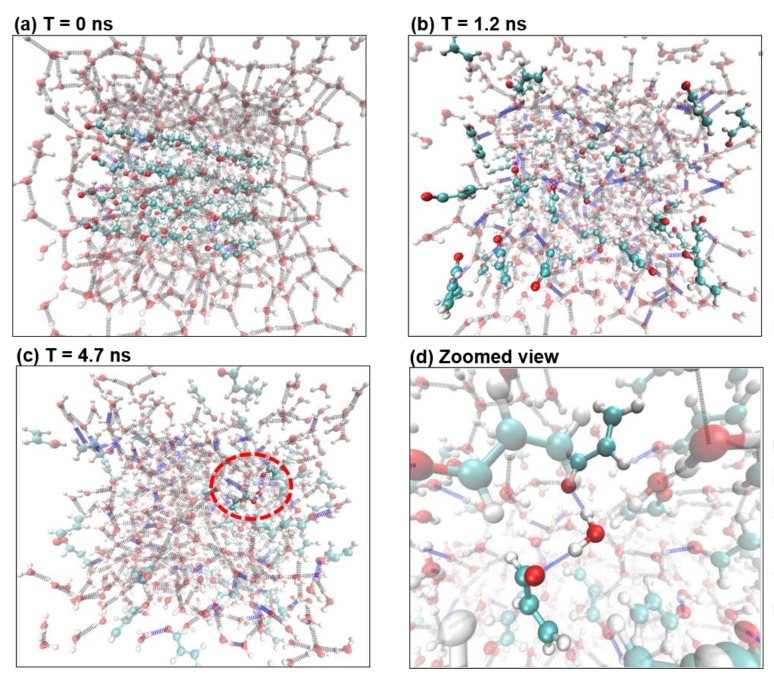
Acrolein molecules solvated in water. (**a**) *T* = 0 ns; (**b**) *T* = 1.2 ns, where more hydrogen bonds formed between water and acrolein; (**c**) *T* = 4.7 ns; (**d**) The circled portion from (**c**), showing two acrolein molecules connected by a water molecule through hydrogen bonds (the rest of the molecules are set to be transparent). Color scheme: Hydrogen in white, oxygen in red, carbon in cyan, blue for hydrogen bonds between waters and acrolein, and grey for hydrogen bonds between water molecules. In (**a**–**c**), the water molecules are set to be transparent for a better view of the acrolein compounds.

Figure 8 shows the dynamics of 72 acrolein molecules in the bath of 800 water molecules at different instants from an initial homogenous spatial arrangement (Figure 8a)), a video of the acrolein binding process was also provided as a supplementary material. The water molecules were set to be transparent for a better view of the acrolein. The water-acrolein bonds are shown in green, and new hydrogen bonds (dotted blue lines) started to form between water and acrolein molecules (Figure 8b) and quickly reached a statistically stable state at *T* = 4.7 ns (Figure 8c). Both acrolein-water and acrolein-water-acrolein binding were observed to be connected by hydrogen bonds between a hydrogen-oxygen pair. One example of acrolein-water-acrolein binding is highlighted in Figure 8d, while the rest of molecules are shown as transparent. The molecular binding was also quantified. There were 35 newly formed acrolein–water hydrogen bonds out of the 72 initially free acrolein molecules (48.6%), and this number is statistically constant from *T* = 4.7 ns. Among them, there were seven acrolein-water-acrolein compounds (10%). Interestingly, acrolein–acrolein bonds were not observed, indicating a higher affinity of acrolein to water molecules than to acrolein itself. Moreover, most acrolein-water bonds were also accompanied by an additional water-water bond, forming an acrolein-water-water structure. All this binding increases the compound size and reduces the compound’s effective diffusivity to varying degrees.

**Figure 9:**
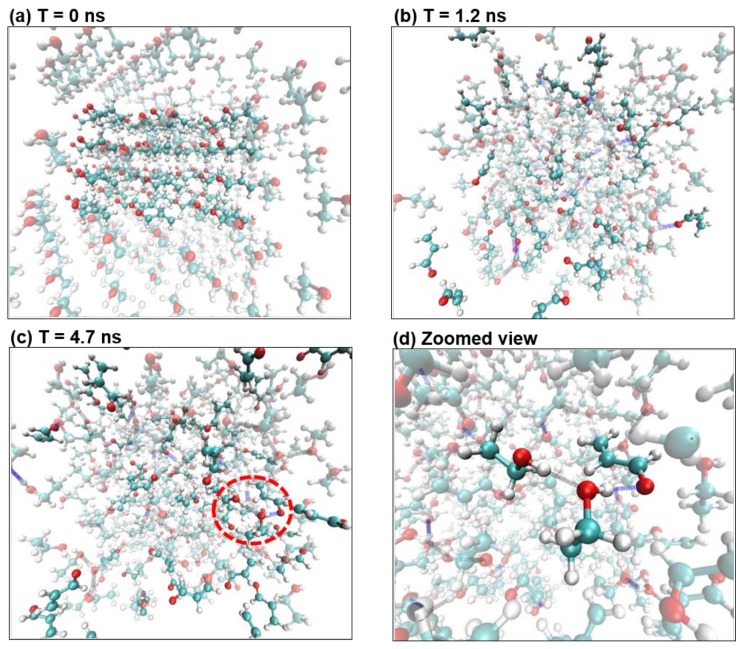
Acrolein molecules solvated in ethanol. In (**a**–**c**), the ethanol molecules are set to be transparent for a better view: (**a**) *T* = 0 ns; (**b**) *T* = 1.2 ns; (**c**) *T* = 5.4 ns; (**d**) the circled portion in (**c**), showing one acrolein connected to two ethanol molecules through hydrogen bonds. Color scheme: hydrogen in white, oxygen in red, carbon in cyan, blue for hydrogen bonds between waters and acrolein, and grey for hydrogen bonds between water molecules.

Effects of the medium polarity on molecular binding were evaluated by immersing acrolein in ethanol molecules. The dynamics of the acrolein–ethanol field are displayed in Figure 9a–c at different instants. Similarly, ethanol molecules are set as transparent to highlight the acrolein molecules. Figure 9d shows one binding example, in which one acrolein is connected to two ethanol molecules through hydrogen bonds. The hydrogen bonds connecting acrolein–ethanol are shown as blue whereas the bonds connecting ethanol-ethanol are grey. Quantitatively, there were 15 acrolein–ethanol bonds out of 72 initially free acrolein molecules (20.8%), which is much lower than the percentage of 48.6% in water.

**Figure 10:**
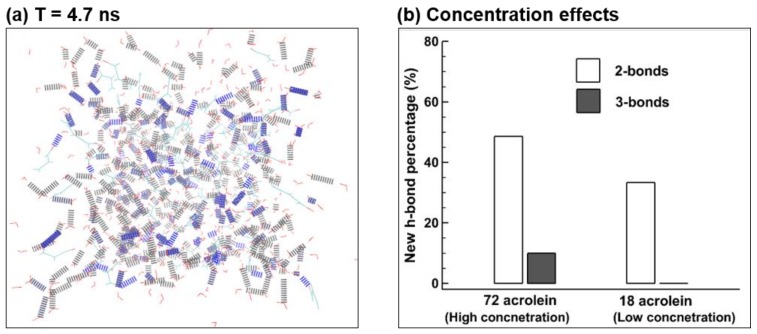
Concentration effects: (**a**) Hydrogen bonds at *T* = 4.7 ns and (**b**) percentage of new hydrogen bonds in two different acrolein concentrations. Color scheme: hydrogen in white, oxygen in red, carbon in cyan, blue for hydrogen bonds between waters and acrolein, and grey for hydrogen bonds between water molecules.

Effects of the acrolein concentration on the binding probability were evaluated by reducing the number of initial acrolein molecules to 18. The statistic of molecular binding was quantified at *T* = 4.7 ns for each case and the binding fractions are compared in Figure 10 in terms of acrolein–water and acrolein-water-acrolein compounds. It is shown that there are 33.3% (6 out of 18) newly formed acrolein-water bonds in the low concentration case (18 acrolein molecules) in comparison to 48.6% (35 out of 72) in the high concentration case. Furthermore, no configuration with three hydrogen bonds was observed in the low concentration case, while three-bonds configurations made up 10% in the case of the 72 acrolein molecules.

## 3. Discussion

Multiscale modeling and simulations were conducted to gain a better understanding of an interesting experimental observation that the deposition of acrolein in rat airways decreases at higher concentrations. It was hypothesized that, in addition to the limit of the Michaelis-Menten (MM reaction rate, intermolecular interactions between acrolein and water molecules might also contribute to the decreased deposition by reducing the effective vapor diffusivity. Both computational fluid dynamics (CFD), chemical species transport, and molecular dynamics (MD) simulations were conducted. Instant reaction was assumed in this study in order to isolate the potential effects of intermolecular interactions (binding). Results demonstrated that molecular binding indeed occurred between acrolein and water molecules, which decreased the effective vapor diffusivity and vapor deposition. A higher percentage of acrolein-water hydrogen bonds was observed at higher acrolein concentrations, in the form of both one-hydrogen and of two-hydrogen bonded configurations. Similarly, molecular binding for acrolein is possible in human respiratory airways, which will cause lower deposition fractions at higher ambient concentrations.

MD simulation results of this study compare favorably with the quantum mechanics study by Georg et al. [26]. Statistically, 48.6% of acrolein compounds have one hydrogen bond and about 10% have two hydrogen bonds. Acrolein compounds with three hydrogen bonds were not observed in this study. In comparison, the statistics of hydrogen bonds between acrolein and water predicted in [26] were 60% having one hydrogen bond, 20% having two hydrogen bonds, and 1% having three hydrogen bonds. The slight discrepancies may be attributed to the force field difference adopted between these two studies, as more recent data on acrolein [27] was used in this study.

To answer the question raised in the beginning of this paper regarding the possible phase of acrolein in the airways, it is helpful to revisit the difference between gas vapor and liquid droplets of water. Under atmospheric pressure, the boiling point of water is 100 °C. In principle, when the partial pressure is below the saturation pressure, water molecules exist in the gas (or vapor) phase, but in the air, the liquid phase is negligible or nonexistent. A fundamental question is that, even in this scenario, do all water molecules exist as free molecules? Or, is it possible that several water molecules bond together (statistically) as an agglomerate, but that the agglomerate size is still not large enough to be considered liquid droplets? This question is especially relevant to the high polarity of the water and/or acrolein molecules, considering the fact that different physical properties may result from the intermolecular interactions. MD simulation results in this study showed that binding between acrolein and water molecules indeed occurred, in terms of either one-hydrogen or two-hydrogen bonds. Statistically, because molecular binding–breaking is a dynamic process, larger compounds will be formed, which increases the compound inertia and reduces the chemical diffusivity.

The finding that chemical diffusivity may vary with concentration has important implications for the inhalation dosimetry of highly polar chemicals. A greater diffusivity will not only lower the deposition rate, but also change the deposition distribution of inhaled chemicals. The local or regional deposition of inhaled aerosols is a more relevant factor in developing a dose-response relationship than the overall deposition rate. Understanding the cell responses from the cellular (or even intracellular) dose, as opposed to the nominal dose, is more likely to disclose the mechanism underlying the adverse health effects from exposure to such toxicants. On the other hand, using a concentration-invariant diffusivity in the computational inhalation dosimetry may not accurately predict the total and local deposition rates for high-polarity chemicals to different degrees at different concentrations.

One example worthy of reconsideration is the acrolein dosimetry from cigarette smoking. Current cigarette smoke tests typically use one of three standardized smoking machine regimes: ISO (the International Organization for Standardization), MDPH (the Massachusetts Department of Public Health), and HC (Health Canada) [28]. These three regimes differ in their puff volume, duration, frequency, and intervals to give different inhalation concentrations, with those of HC being approximately five times those of ISO [28]. As expected, the deposition fraction of acrolein under an MDPH smoking condition is lower than under an ISO condition. In other words, the tissue dose increases at a slower rate than the exposure concentration, and the linear assumption that has generally been practiced in dose-response analyses is no longer valid unless the tissue or cellular dose is identified.

Reasons for the increasing fraction of acrolein–water bonds at higher acrolein concentrations are speculated below. When an acrolein molecule was put into an initially stable bath of water molecules, a disturbance was generated both spatially and dynamically. Water molecules exhibit greater affinity for each other than for acrolein molecules, as evidenced by the higher negative charge of the oxygen atom (O) in water (−0.820 e) than in acrolein (−0.507 e) [27]. The disturbance caused by one acrolein molecule, in combination with the acrolein polarity, may not be strong enough to break the existing hydrogen bonds and form new acrolein–water hydrogen bonds. The disturbance, however, increases when more acrolein molecules are introduced into the same bath of water molecules, exerting more stress on the existing hydrogen bonds and making them more vulnerable to be broken. As a result, the probability of forming new acrolein-water bonds increases. As more acrolein molecules are introduced, the chance of forming acrolein–water–acrolein complexes also increases (Figure 10).

Some assumptions may limit the applicability of the results of this study. Assumptions of CFD simulations include a complete absorption rate (*c_wall_* = 0), steady flows, rigid walls, no phase change, and a small cohort of human airway models. Complete absorption was assumed to isolate the effect of chemical diffusivity (or, equivalently, particle diameter). Vapor absorption into tissue is often limited by the maximum Michaelis–Menten metabolism rate, which is proportional to the enzyme concentration in the tissue [29]. To account for the decreased deposition rates at higher concentrations [9,10], the required molecule compound with the assumption of *c_wall_* = 0 should be 2.1 nm at 2 µg/L, 4.8 nm at 10 µg/L, and 6.2 nm at 20 µg/L (Figure 5). The latter two cases are much larger than the possible sizes of new hydrogen-bonded acrolein compounds. Therefore, molecular binding can only explain part of the deposition decrease, while the limited Michaelis–Menten metabolism rate is still the major reason. The influences of tidal breathing [30,31,32], dynamic airways [33,34,35,36], polydisperse aerosols [37,38], hygroscopic growth [39,40,41], electric charges [42,43], and intersubjective variability [44,45,46] on acrolein deposition are also important and should be considered in future studies. Imaging techniques such as phase-contrast MRI (magnetic resonance imaging) with hyperpolarized ^3^He can be used to visualize respiratory flows [47,48]. It is acknowledged that MD and CFD simulations were conducted separately, without coupling, in this study, as two distinct methods for understanding the experimentally observed concentration-dependent acrolein deposition in rats.

## 4. Materials and Methods

### 4.1. Rat Airway Model

An anatomically accurate rat airway model was used to numerically predict the deposition of acrolein in the upper respiratory tract. This rat nasal airway surface model was previously reconstructed by Corley et al. [21] from MR imaging at 0.125 mm resolution of a 10-week-old Sprague Dawley rat (weight 300 g). The computational domain includes the nose, trachea, and lungs that extend to approximately the ninth bifurcations, as shown in Figure 1. The airway surface area of this model is 12.42 cm^2^ and the volume is 0.248 cm^3^. Similarly, a surface area of 13.73 cm^2^ and an airway volume of 0.353 cm^3^ were reported in a male Sprague–Dawley rat of similar weight by Menache et al. [49]. A comparison of nasal airway sizes between rats and humans in different regions can be found in [20].

### 4.2. Computational Flow-Vapor Transport Models

Steady breathing conditions and incompressible, isothermal airflows were assumed for all test cases. The inhalation flow rate was 430 mL/min, which is typical for rat normal breathing [21]. This gave a velocity of 2.32 m/s at the nostrils. A uniform velocity profile (*u_in_* = 2.32 m/s) was specified at the inlet. Based on a hydraulic diameter of 1.5 mm and a velocity of 4 m/s at the throat (Figure 1a), the maximum Reynolds number was around 360 at the throat, indicating a laminar flow regime in the airway. Zero pressure was specified at the outlets. A well-tested low-Reynolds-number *k-ω* turbulence model was implemented to resolve the flow field based on its capacity to provide an accurate solution for transitional and laminar flow as the turbulent viscosity approaches zero [50]:

(1) $$\frac{\partial {\bar{u}}_{i}}{\partial x_{i}}=0$$

(2) $$\frac{\partial {\bar{u}}_{i}}{\partial t}+{\bar{u}}_{j}\frac{\partial {\bar{u}}_{i}}{\partial x_{j}}=-\frac{1}{\rho }\frac{\partial p}{\partial x_{i}}+\frac{\partial }{\partial x_{j}}\left[\left(\nu +\nu_{T}\right)\left(\frac{\partial {\bar{u}}_{i}}{\partial x_{j}}+\frac{\partial {\bar{u}}_{j}}{\partial x_{i}}\right)\right]$$

where ${\bar{u}}_{i}$ is the time-averaged velocity in three coordinate directions, i.e., *i* = 1, 2, and 3, *p* is the time-averaged pressure, *ρ* is the fluid density, and *ν* is the kinematic viscosity. Transport equations governing the turbulent kinetic energy (*k*) and the specific dissipation rate (*ω*) are:

(3) $$\frac{\partial k}{\partial t}+{\bar{u}}_{j}\frac{\partial k}{\partial x_{j}}=\tau_{ij}\frac{\partial {\bar{u}}_{i}}{\partial x_{j}}-\varepsilon_{k}+\frac{\partial }{\partial x_{j}}\left[\left(\nu +0.5\nu_{T}\right)\left(\frac{\partial k}{\partial x_{j}}\right)\right]$$

(4) $$\frac{\partial \omega }{\partial t}+{\bar{u}}_{j}\frac{\partial \omega }{\partial x_{j}}=\frac{13}{25}\frac{\omega }{k}\tau_{ij}\frac{\partial {\bar{u}}_{i}}{\partial x_{j}}-\varepsilon_{\omega }+\frac{\partial }{\partial x_{j}}\left[\left(\nu +0.5\nu_{T}\right)\left(\frac{\partial \omega }{\partial x_{j}}\right)\right]$$

In the above equations, $\tau_{ij}$ is the shear stress tensor, $\varepsilon_{k}$ and $\varepsilon_{\omega }$ represents the dissipation of *k* and ω, respectively. This model has been shown to be able to model laminar-to-turbulent transitions in human nasal airways [51,52]. The vapor uptake process consists of three steps: transport of inhaled vapor to the air-tissue interface, mass transfer across the tissue, and diffusion or perfusion of the tissue-borne species away from the tissue. The mass transport relation governing the convective-diffusive motion of vapor in the airflow can be described on a mass fraction basis as:

(5) $$\frac{\partial C_{a}}{\partial t}+\frac{\partial \left(u_{j}C_{a}\right)}{\partial x_{j}}=\frac{\partial }{\partial x_{j}}\left[\left(D_{a}+\frac{\nu_{T}}{Sc_{T}}\right)\frac{\partial C_{a}}{\partial x_{j}}\right]$$

(6) $$D_{a}=\frac{k_{B}TC_{c}}{3\pi \mu d_{p}}$$

In the above equations, *C_a_* represents vapor concentration in the airflow with the unit of mg/m^3^, *Sc_T_* is the turbulent Schmidt number, taken to be 0.9 [53], *k_B_* = 1.38 × 10^−16^ cm^2^ g/s is the Boltzmann constant, and *C_c_* is the Cunningham correction factor. $D_{a}$ (m^2^/s) is the molecular or Brownian diffusion coefficient of the species in the air, which depends on the size of the compound (*d_p_*), as shown in Equation (6). The inlet concentration was specified as 0.1% and the outlet condition as specified as zero flux. The local mass flux to the wall is computed as:

(7) $${\dot{m}}_{l}=-\rho A_{l}\left(D_{a}+\frac{\nu_{T}}{Sc_{T}}\right){\frac{\partial c}{\partial n}|}_{wall}$$

where the subscript *l* denotes the local region, *A_l_* is the local area, and *n* is the wall normal direction. This model has been demonstrated to accurately predict transport and deposition for vapor species [54,55] in human airways.

Computational mesh for the rat nose models was generated using ANSYS ICEM CFD (Ansys, Inc., Canonsburg, PA, USA). Mesh sensitivity analysis was conducted to ensure a grid-independent deposition rate. The final mesh had 3.8 million cells [20]. ANSYS Fluent (Ansys, Inc.) was used to solve the airflow and vapor transport. The uptake of acrolein into the circulation system can be accounted for by a two-layer model [56,57]. The mucus and epithelium are lumped together as one layer and the vascularized sub-epithelial blood vessels as the second layer. The apical side tissue interfaces with the airway lumen and the basal side interfaces with the blood. A coupled diffusion equation can be analytically solved to determine the wall concentration of acrolein *C_a_|_n=_*_0_ as a function of the acrolein concentration in the airway lumen *C_a_* [56,58]:

(8) $$C_{a}{|}_{n=0}=\frac{C_{a}}{1+\kappa \cdot ds}$$

(9) $$\begin{array}{cc} \kappa =\frac{\lambda_{ta}}{D_{a}}\left(\frac{D_{t}}{H_{t}}+\frac{\gamma }{2}\right), & \gamma =H_{t}(k_{f}+\frac{V_{\max}C}{K_{m}+0.5\lambda_{ta}C_{a}{|}_{n=0}}) \end{array}$$

where *ds* is the normal direction from the wall surface of the first layer cells, *λ_ta_* is the tissue-air partition coefficient, *k_f_*, is the first-order reaction rate, *V_max_C* is the saturable reaction rate per unit volume of the tissue, and *K_m_* is the Michaelis–Menten constant [57]. In this study, complete absorption and quick reaction were assumed (*C_a_|_n=_*_0_ = 0) to isolate the effect of vapor diffusivity.

### 4.3. Molecular Dynamic (MD) Simulations

The open-source package GROMACS 4.6 (GROningen MAchine for Chemical Simulations, Uppsala, Sweden) [59] was used for the MD simulations of acrolein among water and ethanol molecules. Visual Molecular Dynamics (VMD) was used to visualize the simulation results [60]. The non-bonded potentials were calculated with a Lennard–Jones potential for van der Waals and with Coulomb’s law for the electrostatic potentials. More details of the force field for acrolein can be found in Malde et al. [27]. The system was coupled to an isotropic pressure coupling of 1 atmosphere with a homogeneous compressibility of 4 × 10^−5^ in all three directions. The temperature was specified as 310.5 K (body temperature). The velocities of the different molecules (water, ethanol, and acrolein) were generated subject to Maxwellian distribution [61] and were scaled with a coupling constant of 0.1 ps. The algorithms for pressure and temperature control are discussed in Allen and Tildesly [62]. A time step of 3 fs was used with constraints on the bond lengths within the acrolein and the water geometry. A twin cutoff scheme was employed for the non-bonded interactions, with *R*_cutoff_ being 1.0 nm for the Coulomb potential and *R*_cutoff_ being 1.2 nm for the van der Waals interaction calculation [63]. Particle-mesh Ewald (PME) summation was applied to calculations of the long-range electrostatic interactions. The Simple Point Charge (SPC) water model was used with a charge of –0.82 *e* on the oxygen atom, and charges of 0.41 *e* on each of the hydrogen atoms [27]. Two systems were studied: one with 72 acrolein molecules solvated by 708 SPC water molecules in a cubic box with a side length of 3 nm, and the other with 72 acrolein molecules solvated by 206 ethanol molecules in the same sized cubic box. The time step size was 0.5 fs (i.e., 0.5 × 10^−15^ s), with about 10–12 million steps (5–6 ns) till the system became stable.

## 5. Conclusions

In summary, this study investigated intermolecular interactions and their effects on acrolein deposition in a rat airway model using MD and CFD simulations. Specific findings from this study can be summarized as follows:

1. Acrolein–water compounds were predicted to have either one (i.e., acrolein–water) or two (i.e., acrolein-water-acrolein) hydrogen bonds.
2. The fraction of hydrogen-bonded acrolein compounds over free acrolein molecules increased with larger acrolein concentrations and higher media polarity.
3. The decreased acrolein-compound diffusivity due to molecular binding lowered the total deposition rate and altered the deposition distribution in the rat airway.
4. Molecule binding cannot explain the entire decrease in acrolein uptake that had been observed experimentally in rats, and acts as a secondary mechanism contributing to the deposition decrease of chemicals with high polarity.
5. Consideration of concentration-dependent diffusivities is recommended in inhalation dosimetry predictions for improved accuracy.

## Supplementary Materials

Supplementary materials can be found at http://www.mdpi.com/1422-0067/19/4/997/s1.
